# The mutation profile of differentiated thyroid cancer coexisting with undifferentiated anaplastic cancer resembles that of anaplastic thyroid cancer but not that of archetypal differentiated thyroid cancer

**DOI:** 10.1007/s13353-020-00594-0

**Published:** 2020-11-22

**Authors:** Justyna Mika, Wojciech Łabaj, Mykola Chekan, Agata Abramowicz, Monika Pietrowska, Andrzej Polański, Piotr Widłak

**Affiliations:** 1 Faculty of Automatic Control, Electronics and Computer Science, Silesian University of Technology, Gliwice, Poland; 2 Maria Skłodowska-Curie National Research Institute of Oncology, Gliwice Branch, Gliwice, Poland

**Keywords:** Cancer evolution, Concurrent cancers, Mutation profiles, Next-generation sequencing, Thyroid cancer

## Abstract

Differentiated thyroid cancer (DTC) has one of the lowest cancer mutational burdens, while anaplastic thyroid cancer (ATC) has a much higher mutation frequency. A fraction of ATC has an associated differentiated component, which suggests the coevolution of both cancers. Here, we aimed to compare mutation frequency in coexisting ATC and DTC diagnosed concurrently in the same thyroid gland (3 cases) as well as in archetypal DTC and ATC alone (5 cases each). Single-nucleotide variations (SNV) and copy number variations (CNV) were analyzed in each case based on the next-generation sequencing data. We found a similar extent of mutational events, both SNV and CNV, in undifferentiated and differentiated components of thyroid cancers coexisting in one patient. The magnitude of these mutations was comparable to the level of mutations observed in ATC alone; yet, it was much higher than in archetypal DTC. This suggested that, despite histopathological features of differentiated tumors, molecular characteristics of such cancers coexisting with ATC and archetypal DTC could be significantly different. Pairwise comparison of mutational profiles of coexisting cancers enabled assumption on the possible evolution of both components, which appeared distinct in 3 analyzed cases. This included independent development of ATC and DTC diagnosed concurrently in two lobes of the same thyroid, as well as the development of anaplastic and differentiated cancer from the common ancestor that putatively gained a key driver mutation (*BRAF*^V600E^ or *KRAS*^Q61R^), which was followed either by early or late molecular separation of both cancers.

**Supplementary Information:**

The online version contains supplementary material available at 10.1007/s13353-020-00594-0.

## Introduction

Thyroid cancer represents a wide spectrum of malignancies, among which the most frequent are papillary thyroid carcinomas (PTC) and follicular thyroid carcinomas (FTC), collectively termed “differentiated thyroid carcinomas” (DTC), which generally have low mortality and high curability (Siegel et al. 2015). On the other hand, undifferentiated anaplastic thyroid carcinoma (ATC), though very rare, is very aggressive and nearly universally fatal (Chiacchio et al. 2008). Epidemiological, clinical, and pathological evidence suggest that ATC may arise de novo, but in most cases, it develops from the transformation of preexisting or coexisting DTC (Venkatesh et al. 1990; Wiseman et al. 2003). Importantly, it has been reported that a fraction of ATC has an associated differentiated component (Xu et al. 2020). The intra-tumoral evolution of ATC from DTC is supported by similarities of the genetic fingerprint of coexisting ATC and DTC, including the concordance of the driver mutation status of both components (Quiros et al. 2005; Ragazzi et al. 2020). Interestingly, a few recent genomics studies compared global mutational profiles of coexisting (concurrent) ATC and DTC (Capdevila et al. 2018; Dong et al. 2018; Ragazzi et al. 2020), which supported both hypothetical models of ATC development, i.e., de novo development from pre-cancerous lesions independent of coexisting DTC or sequential evolution of ATC from preexisting DTC.

DTC is a tumor with a low mutational burden, which was revealed by a pan-cancer study based on the Cancer Genome Atlas data (CGAR Network 2014). This usually harbors only a single driver gene alteration, mostly point mutations in *BRAF* and *RAS* family genes as well as fusions involving *RET* (frequency of other potential drivers is much lower) (Song and Park 2019). On the other hand, genetic instability is usually associated with aggressive undifferentiated cancers, and ATC showed a high mutational burden. Moreover, though *BRAF* and *RAS* remained frequently mutated in ATC, several additional drivers were detected, including *TP53* (Xu et al. 2020; Pozdeyev et al. 2018; Yoo et al. 2019). It is worth noting, however, that in the case of DTC coexisting with ATC, a higher number of somatic mutations than in the undifferentiated component could be observed (Dong et al. 2018). This suggested that differentiated cancers coexisting with ATC could have atypical molecular features, distinct from “archetypal” DTC. Here, we aimed to follow this observation and compared the mutational profiles of archetypal DTC (patients with PTC alone) to “atypical” DTC coexisting with ATC in the same gland as well as mutational profiles of coexisting ATC with ATC alone. In parallel, the mutation profiles of coexisting differentiated and undifferentiated cancers were directly compared that enabled conclusions about their hypothetical evolution.

## Results

Thirteen patients were involved in the study, which included three patients with coexisting ATC and DTC, five patients with ATC alone, and five patients with DTC alone (Table 1). DNA isolated from formalin-fixed paraffin-embedded tissue (FFPE) specimens was analyzed by the exome next-generation sequencing (with sequencing depth > 100-fold), and obtained mutation profiles normalized against the individual reference of normal thyroid were compared among samples (detailed description of the methodology is provided in the supplementary file “Materials and Methods”).

**Table 1:** Description of the clinical material. Postoperative tissue collected during thyroidectomy and stored as formalin-fixed paraffin-embedded material was used. Tissue material was re-inspected by an experienced pathologist before the study; the selected regions of interest (ROI) contained at least 80% of cancer tissue (small amounts of normal thyroid, muscles, and connective tissue could be also present). Moreover, for each patient, normal thyroid reference was collected from a tissue distant from the cancer ROI that showed no marks of any pathology

Patient ID	Sex	Age	Histopathology	pTNM (8th ed.)
ATCx-1/DTCx-1 (case 1)	F	76	Two tumors present simultaneously: undifferentiated anaplastic carcinoma (right lobe, 4 cm) and differentiated papillary carcinoma (left lobe, 1.5 cm)	pT3aN1a (ATC); pT1bN0 (PTC)
ATCx-2/DTCx-2 (case 2)	M	68	Undifferentiated anaplastic carcinoma with areas of differentiated papillary carcinoma (8 cm)	pT3bN1a
ATCx-3/DTCx-3 (case 3)	M	60	Undifferentiated anaplastic carcinoma with areas of invasive differentiated follicular carcinoma (11 cm)	pT3bN1b
ATC-1	F	72	Undifferentiated (anaplastic) thyroid carcinoma (3.5 cm)	pT2N1b
ATC-2	M	35	Undifferentiated (anaplastic) thyroid carcinoma (4 cm)	pT3bN1b
ATC-3	F	43	Undifferentiated (anaplastic) thyroid carcinoma (5 cm)	pT3aNx
ATC-4	F	66	Undifferentiated (anaplastic) thyroid carcinoma (6 cm)	pT3bNx
ATC-5	M	43	Undifferentiated (anaplastic) thyroid carcinoma (8 cm)	pT3aN0
DTC-1	M	68	Papillary thyroid carcinoma, follicular variant (1.3 cm)	pT1b(m)Nx
DTC-2	F	25	Papillary thyroid carcinoma, follicular variant (1.5 cm)	pT1bNx
DTC-3	F	36	Papillary thyroid carcinoma, follicular variant (1.3 cm)	pT1bN1a
DTC-4	F	28	Papillary thyroid carcinoma, classical variant (3.9 cm)	pT2N1b
DTC-5	F	42	Papillary thyroid carcinoma, follicular variant (2.2 cm)	pT2N1a

**Fig. 1:**
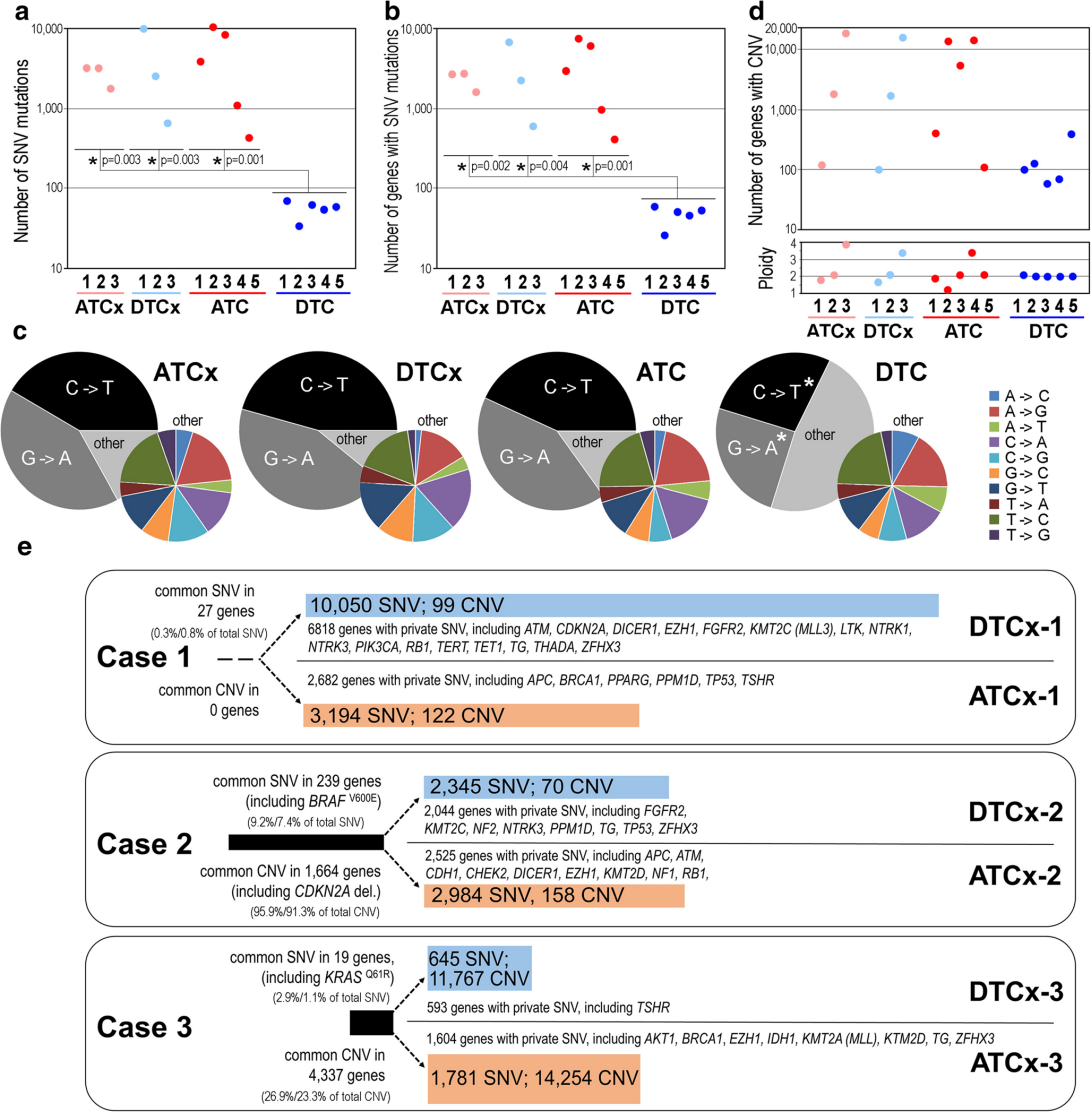
Comparison of mutation profiles in DTC and ATC revealed by the exome next-generation sequencing. The total number of SNV alterations (**a**) and genes with SNV (**b**) in samples of coexisting undifferentiated and differentiated cancers (ATCx and DTCx, respectively), ATC alone, and DTC alone (numbers correspond to individual patients); *y* axes in the log scale; *p* values refer to pairwise comparisons between DTC and another group. **c** The relative contribution of different base substitutions, pie charts represent the mean percentage of each substitution, smaller color-coded charts represent “other” changes depicted in larger charts (all “other” substitutions are summed up to 100%), and asterisks mark the significance of differences (*p* < 0.05) between DTC and all other groups when compared pairwise. **d** The number of genes with CNV (upper graph; *y* axis in the log scale) and the estimated ploidy (bottom graph) in compared groups (description as for **a**). For all panels, the significance of differences was estimated by the Kruskal-Wallis rank ANOVA test followed by the Conover-Iman *post hoc* test. **e** Pairwise comparison of mutation profiles in ATCx and DTCx coexisting in one gland (cases 1–3). Root mutations are represented by black bars; branch mutations specific for differentiated and undifferentiated components are represented by blue and salmon bars, respectively; and common mutations are presented also as a percentage of all mutations detected in a given tumor (DTCx/ATCx). Listed are putative driver genes according to references quoted in the text (CGAR Network 2014; Pozdeyev et al. 2018; Song and Park 2019; Yoo et al. 2019; Xu et al. 2020)

In the first step, we compared the number of different types of mutations among four subsets of samples: ATCx (i.e., ATC coexisting with DTC), DTCx (i.e., DTC coexisting with ATC), ATC, and DTC. A very high number of total single-nucleotide variations (SNV) were noted in the first three subsets (median values were 3221, 2588, and 3935, respectively). In marked contrast, the average number of SNV in DTC was markedly lower (median 59) (Fig. 1a, Supplementary Table S1). A similar pattern of differences was observed in the total number of genes with SNV mutations (median numbers were 2709, 2283, 2959, and 51 genes in ATCx, DTCx, ATC, and DTC, respectively) (Fig. 1b, Supplementary Table S2). When the relative contribution of different base substitutions was compared, we found that C → T and G → A transitions dominated over other changes in all cases; yet, their relative contribution was markedly lower in the DTC subset than in the other three subsets (Fig. 1c, Supplementary Table S3). However, when less frequent base substitutions were analyzed, their relative contributions were similar among the four subsets. Nevertheless, when two dominating substitutions were removed from the analysis, large differences in the number of remaining SNV persisted between samples of DTC alone versus other cancers (median number of such variations was 433, 212, 309, and 28 for ATCx, DTCx, ATC, and DTC, respectively). Then, we looked for copy number variations (CNV) (i.e., genes with copy number ≠ 2). In this case, large variability in the CNV was noted within every subset, which corresponded to the overall ploidy estimated in each sample (Fig. 1d, Supplementary Table S4). Noteworthy, coexisting ATCx-3 and DTCx-3 showed amplification of large chromosome fragments (estimated ploidy 3.9 and 3.4, respectively). Also, marked amplifications (ATC-4, ploidy 3.4) and deletions (ATC-2, ploidy 1.2) of chromosome fragments were noted in the subset of ATC. The number of CNV was the lowest in the subset of DTC (their estimated ploidy was about 2.0). The median number of CNV in compared subsets was 1822, 1734, 5379, and 102 in ATCx, DTCx, ATC, and DTC, respectively. We concluded that the number of SNV and CNV mutations in samples of differentiated thyroid cancer coexisting with anaplastic cancer (i.e., DTCx) was similar to that in undifferentiated cancers (ATCx and ATC) yet markedly higher than in archetypal DTC.

In the second step, we pairwise compared mutations detected in regions of undifferentiated and differentiated cancer coexisting in the same thyroid gland (cases 1, 2, and 3). In each case, a subset of mutations common for both regions (i.e., root mutations) and two subsets of mutation unique for either region (i.e., branch or private mutations) was identified (Fig. 1e), which enabled conclusions about the hypothetical evolution of coexisting cancers. For case 1, a low number of root mutations was noted, which included no putative driver mutations. In marked contrast, a large number of private SNV was noted in both coexisting tumors (that included several putative drivers). Moreover, both tumors showed a low number of CNV. For case 2, a relatively large number of root mutations were noted, which included putative driver mutation *BRAF*^V600E^. Moreover, large subsets of branch SNV were noted, which were associated with several putative drivers. Furthermore, in the common subset, there were 1664 genes with CNV; yet, only a few genes with CNV were unique for either branch. For case 3, only a few root SNV were found; yet, this subset included putative driver mutation *KRAS*^Q61R^. Moreover, numerous CNV were common for both coexisting tumors. Further CNV were unique for both branches, which indicated facilitated chromosome amplification phenotype in both coexisting cancers. Moreover, a relatively large number of unique SNV was noted in the undifferentiated branch, including a few putative drivers (markedly lower number of private SNV was observed in the differentiated branch).

## Discussion

The pairwise comparison of mutational profiles in coexisting ATC and DTC enables a conclusion on the hypothetical evolution of these cancers. Previously, Capdevila et al. (2018) compared such cancers in three patients and found only a few root mutations and large subsets of branch mutations with different driver events, which suggested early separation and independent evolution of coexisting components. On the other hand, a similar analysis performed by Dong et al. (2018) in five patients revealed shared driver mutations between concurrent cancers. Moreover, there were 2 cases where root mutations were markedly less frequent than branch mutations and 3 cases with similarly frequent root and branch mutations, which suggested common evolution than earlier or later separation of both components during tumor development, respectively. Here, we included three cases of coexisting ATC and DTC, which analysis revealed three different hypothetical models of the evolution. In case 1 (ATC and PTC diagnosed concurrently in two different lobes), only a few common mutations were noted including none in the known cancer-related genes. On the other hand, several private putative drivers were identified in either cancer (including *CDKN2A*, *RB1*, and *TERT* in PTC and *APC*, *BRCA1*, and *TP53* in ATC). Hence, though the number of common SNV was slightly higher than among unrelated ATC (from 0 to 7 common SNV in pairwise compared samples), the independent development of ATC and DTC could be assumed (possibly from common pre-cancerous lesion). Noteworthy, the number of SNV was higher in the histologically differentiated tumor than in the undifferentiated one in this particular case. In case 2 (ATC with areas of PTC), a relatively high number of SNV common for both regions was noted, including *BRAF*^V600E^ (i.e., the major driver mutation frequently associated with classical pathology of PTC). The frequency of private alterations was similar in both branches, which included further putative drivers (e.g., *TP53*). Moreover, a large number of CNV common for both compartments were observed, including the deletion of one allele of *CDKN2A* (which is a frequent event in ATC); yet, subsequent private CNV were rather rare. Hence, one could assume that both cancers evolved from a common ancestor that gained *BRAF*^V600E^ mutation. A relatively large number of root SNV and generally common CNV indicated the late separation of both branches. In case 3 (ATC with areas of FTC), a low number of common SNV were noted; yet, this included *KRAS*^Q61R^ (i.e., known driver mutation associated with follicular morphology of thyroid cancer). Private SNV were markedly less frequent in the differentiated compartment than in the undifferentiated one and included only a few thyroid cancer-related genes. On the other hand, a large number of common CNV were noted, and both branches acquired further massive CNV that resulted from the duplication of large fragments of chromosomes. Hence, one could assume that both cancers evolved from a common ancestor that gained *KRAS*^Q61R^ mutation and a high ability for chromosome instability. However, a small number of common point mutations and their markedly lower level in the differentiated component could suggest the earlier separation of both branches when compared with case 2. Hence, for each of the three clinical cases with coexisting ATC and DTC reported in this communication, different hypothetical models of cancer evolution could be assumed.

Typical DTC and ATC have significantly different mutational burdens, in general. Hence, potential differences in mutation profiles between archetypal DTC and histologically differentiated tumors coexisting in one gland with ATC appear an intriguing question. Here, we found a generally similar level of point mutations detected in coexisting DTC and ATC, which was comparable with that found in a subset of ATC alone. In marked contrast, the level of point mutations was significantly lower in a subset of archetypal DTC (classical and follicular variants of PTC). In this study, all detected single-base alterations were called irrespective of their putative/potential impact and pathogenicity that enabled a wider characterization of mutational changes; yet, the reported frequency of mutations was markedly higher (approx. an order of magnitude) compared with other studies. However, if SNV with putatively high or moderated impact on a gene function (McLaren et al. 2016) were considered, which returned numbers of single-base substitutions comparable to that reported in previous larger studies (Pozdeyev et al. 2018; Yoo et al. 2019), differences between sample subsets remained very high (median number of high impact alterations was 88, 68, 59, and 0 for ATCx, DTCx, ATC, and DTC, respectively, Supplementary Table 2). Noteworthy, the C → T and G → A transitions dominated in all subsets of samples, which may reflect artifacts observed in DNA purified from the FFPE material (Do and Dobrovic 2015). However, when both artifact-prone substitutions were removed, large differences in the number of remaining SNV persisted among compared subsets. Furthermore, archetypal DTC generally appeared diploid and showed a low extent of CNV. The picture was more complex in the other three subsets, where amplifications and deletions of large chromosome segments were observed and the frequency of CNV was generally higher than in DTC alone. In aggregate, our data indicate that the mutational profile of differentiated thyroid cancers coexisting in one gland with undifferentiated cancer generally resembles that of typical ATC yet is distinct from that observed in typical DTC. This may suggest that thyroid cancers called “differentiated” based on their microscopic morphological features that are prone to transformation into the ATC could be molecularly distinct species compared with archetypal DTC. However, clinical implications of this observation are not clear because, though patients with archetypal DTC have a better prognosis than patients with concurrent DTC and ATC, worse clinical outcomes (e.g., mortality) in the latter group is primarily attributed to the presence of more aggressive undifferentiated component.

## Electronic supplementary material

ESM 1 (PDF 437 kb)

ESM 2 (XLSX 6209 kb)

## Data Availability

The raw NGS data will be available upon request.
