# Supplementary material for: The mutation profile of differentiated thyroid cancer coexisting with undifferentiated anaplastic cancer resembles that of anaplastic thyroid cancer but not that of archetypal differentiated thyroid cancer
Source: J Appl Genet. 2020 Nov 22;62(1):115–20. doi: 10.1007/s13353-020-00594-0 (PMC7822790; doi:10.1007/s13353-020-00594-0)
Supplement: Supplementary file 1 — (PDF 437 kb) [file 13353_2020_594_MOESM1_ESM.pdf]

The mutation profile of differentiated thyroid cancer coexisting with undifferentiated anaplastic cancer resembles that of anaplastic thyroid cancer but not that of archetypal differentiated thyroid cancer.

Supplementary File: **Materials and Methods**

***Clinical material*.** Postoperative tissue collected during thyroidectomy and stored as formalin-fixed paraffin-embedded material was used in the study. Material collected from 13 patients included: 3 patients where coexisting undifferentiated anaplastic thyroid carcinoma and differentiated papillary or follicular thyroid carcinoma was diagnosed concurrently (Case-1, -2, and -3; histopathological pictures are presented below), 5 patients with undifferentiated anaplastic thyroid carcinoma, 5 patients with differentiated papillary thyroid carcinoma (either classical or follicular variant), and (Table 1 presents a detailed description of the clinical material). Thyroidectomy was performed between 2007-2018 at Maria Skłodowska-Curie National Research Institute of Oncology, Gliwice Branch. The study was approved by the Institutional Ethics Committee (Approval No. KB/430-49/12). Tissue material was re-inspected by an experienced pathologist before the study; the selected regions of interest (ROI) contained at least 80% of cancer tissue (small amounts of normal thyroid, muscles, and connective tissue could be also present). Moreover, for each patient, normal thyroid reference was collected from a tissue distant from the cancer ROI that showed no marks of any pathology.

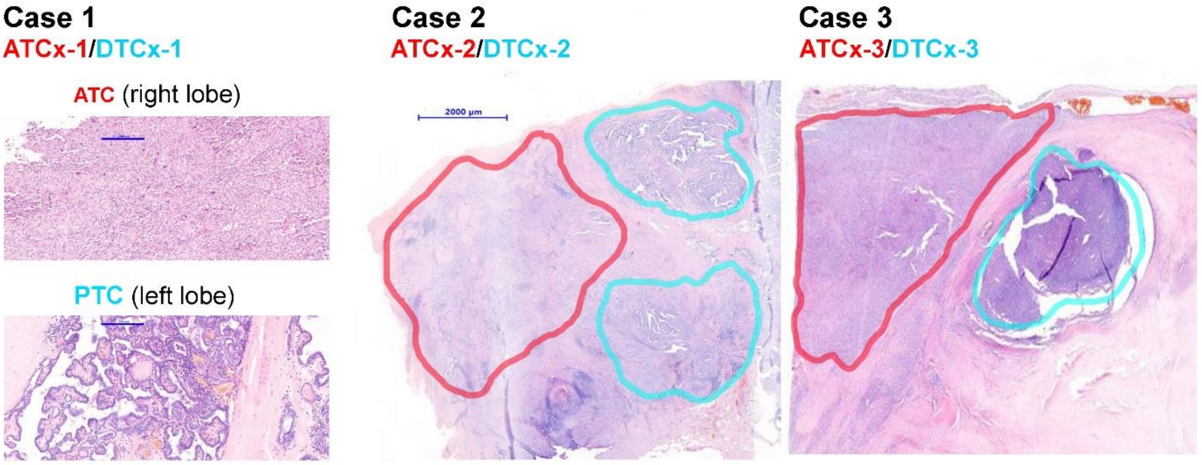

Figure S1. Histopathological picture of undifferentiated and differentiated thyroid carcinomas coexisting in one gland. Case-1 illustrates cancers present simultaneously in two different lobes (showed are two separate tissue sections, scale bare correspond to 200 µm). Case-2 and Case-3 illustrate adjacent ATC (red line) and DTC (blue line) present in the same tissue section (scale bare correspond to 2,000 µm).

DNA sample preparation. Each tissue sample consisted of three cores punched out of the ROI. The extraction of DNA was performed with AllPrep DNA/RNA FFPE Kit (Qiagen, 80234) according to the manufacturer’s protocol. For deparaffinization 500 µL of n-heptan was added to each tube and samples were vigorously vortexed for 10 sec. After 10 minutes of incubation at room temperature, 25 µL of methanol was added and samples were vortexed and centrifuged at 9,000 x g for 2 minutes. The supernatant was completely removed and the pellets were washed once by adding 1 mL of absolute ethanol. After 2 minutes of centrifugation at 20,000 x g the supernatant was removed and pellets were left to dry at 25°C for 10 minutes. Afterward, pellets were resuspended in 150 µL of PKD buffer, and 10 µL of proteinase K was added to each tube. After 15 minutes of incubation at 56°C tubes were placed on ice for another 10 minutes, then centrifuged at 20,000 x g for 15 minutes. The DNA-contained pellets were resuspended in 180 µL of ATL buffer and samples were treated with proteinase K (40 µL per sample) for 1 h at 56°C (with gentle agitation) then for 2 h at 90°C (with no agitation). After cooling to room temperature samples were treated for 2 minutes with 4 µL of RNase A (100 mg/mL), then a premixed solution of buffer AL (200 µL) and absolute ethanol (200 µL) was added to each sample and immediately vortexed. The whole content of the sample was transferred to QIAamp MinElute spin column and centrifuged at 14,000 x g for 1 minute. Then columns were washed successively with 700 µL of buffer AW1, AW2, and absolute ethanol by centrifugation at 14,000 x g for 30 seconds. To completely remove the residual ethanol columns were centrifuged with open lids for an additional 5 minutes at 20,000 x g. Finally, DNA was eluted to clean collection tubes in two steps respectively with 40 and 20 uL of ATE buffer. Each time columns with loaded buffer were incubated for 5 minutes before centrifugation at 20,000 x g for 2 minutes; the volume of the combined eluate was 55 µL. DNA concentration was measured with NanoDrop ND-1000 UV-Vis Spectrophotometer (Thermo Fisher Scientific).

Next-generation sequencing. The exome sequencing was performed by Novogene Genomic Services & Solutions company according to their routine protocol (https://en.novogene.com/). One µg of genomic DNA per sample was used as input material for the DNA library preparation. Sequencing libraries were generated using Agilent SureSelect Human All Exon kit (Agilent Technologies, CA, USA) according to the manufacturer's recommendations with index codes added to each sample. Fragmentation of DNA (to 180-280 bp fragments) was carried out by the hydrodynamic shearing system (Covaris, Massachusetts, USA). Remaining overhangs were converted into blunt ends then after adenylation of 3’ ends adapter oligonucleotides were ligated and DNA fragments with adapters were enriched by PCR. Next, the liquid-phase library hybridization with a biotin-labeled probe was carried out, after which streptomycin-coated magnetic beads were used to capture the exons of genes. Captured libraries were enriched in a PCR reaction to add index tags to prepare for hybridization. Products were purified using AMPure XP system (Beckman Coulter, Beverly, USA) and quantified using the Agilent high sensitivity DNA assay on the Agilent Bioanalyzer 2100 system. Sequencing was performed on Illumina platform with 150 bp paired-end mode and 100x coverage. Burrows-Wheeler Aligner (Li and Durbin 2009), SAMtools (Li et al. 2009) and Picard (Picard 2020) DNA sequencing tools were utilized for mapping paired-end clean reads to the human reference genome GRCh38, for sorting BAM files, and for marking and correcting duplicate reads.

Analysis of NGS data. Detection of somatic mutations was performed by using GATK4- MuTect2 analysis pipeline for paired cancer-normal samples (Benjamin et al. 2019). “Panel of normals” was

created by taking a union of all normal thyroid samples and used as a standard, additional resource for improving mutations calling and their filtration. In the first step, substitution errors caused by FFPE sample preparation were filtered out by using a dedicated "FilterByOrientationBias" option to read orientation artifacts detection and elimination. Next, Mutect2 with default parameters was launched and all mutations, which obtained status (tag) "PASS" were accepted and qualified to the next step (short indels were removed). The final step of filtration performed to obtain more reliable results, involved requirement of coverage of at least 10 reads for both normal and cancer sample, coverage of at least 3 reads on alternative allele and variant allele frequency, VAF $\geq$0.05. In the case of searching for common mutations between co-existing cancers, we required that a somatic mutation pass all filtering steps at least in one of the samples (either ATCx or DTCx), and is detected in the second sample. For each somatic mutation found in the above filtering steps Variant Effect Predictor tool (McLaren et al. 2016) was applied to annotate detected mutations concerning their genomic positions and all potential effects on transcription and translation. However, all point mutations were reported in the current manuscript regardless of their putative/potential impact or "pathogenicity" to illustrate a general picture. Moreover, the Sequenza tool (Favero et al. 2015) was utilized to detect copy number variation segments for each pair of normal-cancer samples. We used our set of parameters to limit the range of the Sequenza searching procedure according to reasonable values confronted with pathologist reports; namely, the ranges 0.8-1.0 were assumed for cellularity and 1.0-4.0 for ploidy.

Statistical analyses. To estimate the significance of differences in the distribution of different base substitutions among four groups of samples the Kruskal-Wallis rank ANOVA test then Conover-Iman test for post hoc pairwise comparisons were used; $p<0.05$ was used as a significance threshold.

Relevant references

Benjamin D, Sato T, Cibulskis K, et al. (2019) Calling Somatic SNVs and Indels with Mutect2, bioRxiv 861054. https://doi.org/10.1101/861054

GATK4 Mutect2 User Guide. https://gatk.broadinstitute.org/hc/en-us/articles/360035889791. Accessed July 2020.

Favero F, Joshi T, Marquard AM, et al. (2015) Sequenza: allele-specific copy number and mutation profiles from tumor sequencing data. Ann Oncol 26:64-70. https://doi.org/10.1093/annonc/mdu479

Li H, Durbin R (2009) Fast and accurate short read alignment with Burrows-Wheeler Transform. Bioinformatics 25:1754-60. https://doi.org/10.1093/bioinformatics/btp324

Li H, Handsaker B, Wysoker A, et al. (2009) The Sequence alignment/map (SAM) format and SAMtools. Bioinformatics 25:2078-9. https://doi.org/10.1093/bioinformatics/btp352

McLaren W, Gil L, Hunt SE, et al. (2016) The Ensembl Variant Effect Predictor. Genome Biol 17:122. https://doi.org/10.1186/s13059-016-0974-4

Picard User Guide. https://broadinstitute.github.io/picard/. Accessed July 2020.
